# Cardioprotection Reloaded: Reflections on 40 Years of Research

**DOI:** 10.3390/antiox14070889

**Published:** 2025-07-18

**Authors:** Pasquale Pagliaro, Giuseppe Alloatti, Claudia Penna

**Affiliations:** 1 Department of Clinical and Biological Sciences, University of Turin, 10043 Orbassano, Italy; claudia.penna@unito.it; 2 National Institute for Cardiovascular Research (INRC), 40126 Bologna, Italy; 3 Uni-Astiss, Polo Universitario Rita Levi Montalcini, 14100 Asti, Italy; giuseppe.alloatti@unito.it

**Keywords:** ischemia/reperfusion injury, oxidative stress, mitochondria, myocardial infarction, NLRP3 inflammasome, reactive oxygen species

## Abstract

Over the past four decades, cardioprotective research has revealed an extraordinary complexity of cellular and molecular mechanisms capable of mitigating ischemia/reperfusion injury (IRI). Among these, ischemic conditioning has emerged as one of the most influential discoveries: brief episodes of ischemia followed by reperfusion activate protective programs that reduce myocardial damage. These effects can be elicited locally (pre- or postconditioning) or remotely (remote conditioning), acting mainly through paracrine signaling and mitochondria-linked kinase pathways, with both early and delayed windows of protection. We have contributed to clarifying the roles of mitochondria, oxidative stress, prosurvival kinases, connexins, extracellular vesicles, and sterile inflammation, particularly via activation of the NLRP3 inflammasome. Despite robust preclinical evidence, clinical translation of these approaches has remained disappointing. The challenges largely stem from experimental models that poorly reflect real-world clinical settings—such as advanced age, comorbidities, and multidrug therapy—as well as the reliance on surrogate endpoints that do not reliably predict clinical outcomes. Nevertheless, interest in multi-target protective strategies remains strong. New lines of investigation are focusing on emerging mediators—such as gasotransmitters, extracellular vesicles, and endogenous peptides—as well as targeted modulation of inflammatory responses. Future perspectives point toward personalized cardioprotection tailored to patient metabolic and immune profiles, with special attention to high-risk populations in whom IRI continues to represent a major clinical challenge.

## 1. Introduction

**Figure 1:**
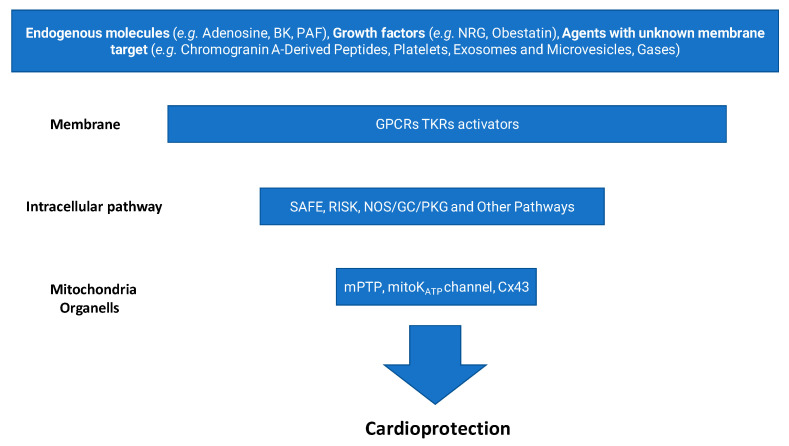
Main steps leading to cardioprotection. (BK, bradykinin; PAF, platelet-activating factor; NRG, neuregulin; GPCR, G-protein coupled receptor; TKR, tyrosine kinase receptor; SAFE, survivor activating factor enhancement; RISK, reperfusion injury salvage kinase; NOS, nitric oxide synthase; GC, guanylate cyclase; PKG, protein kinase G; mPTP, mitochondrial permeability transition pore; mitoK_ATP_, mitochondrial KATP channel; Cx43, connexin 43).

About 20 million deaths were attributed to cardiovascular diseases worldwide in 2020, an increase of 18.7% from 2010 [1]. Myocardial ischemia/reperfusion injury (IRI) induces acute cardiac dysfunction and can be life-threatening, contributing dramatically to these 20 million deaths. Over the past four decades, the quest to protect the heart from IRI has shaped an extraordinary journey in cardiovascular research. What began with the discovery of ischemic preconditioning (IPC) [2] has evolved into a multidimensional field exploring mitochondrial dynamics, redox signaling, kinase cascades, inflammation, and cellular cross-talk, to name a few, e.g., [3,4,5,6,7,8]. Our group has participated in this evolving landscape, dissecting the molecular underpinnings of cardiomyocyte survival, exploring the roles of redox balance and inflammation and proposing novel pharmacological targets and tools, including gasotransmitters, extracellular vesicles, and inflammasome inhibitors. In this prospective review, we start from the discovery of IPC [2], and guided by the “fil rouge” of our research, we develop a brief history of this intriguing research path. Initially demonstrated through preconditioning protocols [2], this strategy has evolved to include postconditioning (applied at the onset of reperfusion) and remote conditioning (triggered by ischemic episodes in distant organs or tissues) [9]. Generally, ischemic conditioning refers to the use of brief, controlled episodes of vascular occlusion and reperfusion to activate endogenous protective mechanisms that can mitigate organ damage caused by more severe ischemic events [10]. These interventions engage rapid signaling pathways involving autocrine/paracrine mediators, like adenosine, nitric oxide, growth factors, and exosomes, as well as kinases, nuclear factors, and mitochondrial effectors [8,11,12] (Figure 1).

A delayed phase of protection, named the second window of protection (SWOP), appearing after 24–48 h, involves the transcription and upregulation of stress-responsive proteins [13,14].

Although preclinical studies across multiple species and organs have consistently shown reductions in tissue injury, clinical translation has been challenging. Benefits in patient-centered outcomes remain limited, likely due to discrepancies between experimental models and real-world patient populations, which often include advanced age, sex-related factors, comorbidities, and polypharmacy. Additionally, many trials have relied on surrogate endpoints rather than hard clinical outcomes [15]. Despite this, some encouraging signals have emerged, especially for remote ischemic conditioning, which may offer cardioprotection in high-risk scenarios, such as delayed reperfusion or hemodynamic instability. Ongoing clinical trials are exploring its potential in more targeted patient groups [NCT04844931 and NCT04813159]. Nevertheless, despite remarkable advances in mechanistic understanding, the translation of cardioprotective strategies from bench to bedside has remained elusive. Clinical endpoints often fail to reflect cellular success, and comorbidities such as diabetes and metabolic syndrome pose significant barriers. However, these challenges have reshaped our vision, calling for a shift toward precision cardioprotection, inclusive experimental models, and multi-centric and integrative approaches that combine immune, metabolic, and electrophysiological insights. This review recapitulates key milestones from our research experience while framing the emerging questions that will guide future efforts toward the necessity of multicenter validation studies. We aim to provide not only a synthesis of past achievements but also a conceptual platform for the next generation of cardioprotective approaches.

## 2. Ischemia/Reperfusion Injury: Cellular and Molecular Basis

While delving into the journey of cardioprotection, it may be helpful to revisit the key mechanisms underlying myocardial IRI, many of which have been elucidated through cardioprotective research itself. Myocardial IRI is a central pathophysiological process in acute myocardial infarction, where, paradoxically, the restoration of blood flow, after an ischemic episode, exacerbates tissue damage [16]. This injury results from a complex interplay of ionic imbalances, oxidative stress, mitochondrial dysfunction, and the activation of various cell death pathways [11]. Cardiomyocyte cell death, particularly through apoptosis, autophagy, ferroptosis, necroptosis, and pyroptosis, is pivotal in myocardial ischemia/reperfusion (IR) injury. These multiple forms of cell death can occur simultaneously in other cell types within the myocardium and micro-vessels. Moreover, they interact with each other and contribute to the complexity of myocardial IRI [9,17].

### 2.1. Ischemia: Pathophysiological Features

Myocardial ischemia results from insufficient oxygen delivery, typically due to impaired coronary blood flow. A concept of myocardial ischemia due to the reduction of coronary blood flow to below 8–10 µL/g per beat with consequences for myocardial electrical, metabolic, contractile, and morphological features has been proposed [18]. Ischemia leads to intracellular acidosis and calcium overload, contributing to impaired electrical coupling through connexon closure and promoting arrhythmogenesis. Locally elevated extracellular K^+^ further disrupts electrical conduction. The Na^+^/H^+^ exchanger (NHE), which extrudes protons in exchange for Na^+^ influx, plays a key role in maintaining intracellular pH but also contributes to intracellular Na^+^ accumulation during ischemia. In turn, this favors Ca^2+^ overload via a reduced forward-mode Na^+^/Ca^2+^ exchange (NCX) [18].

Ischemia also activates ATP-sensitive potassium (KATP) channels, which shorten the action potential and reduce Ca^2+^ influx as an adaptive mechanism to limit energy consumption. This hypoxia-induced shortening is abolished by glibenclamide or the genetic ablation of the Kir6.2 subunit of KATP channels [19]. In contrast, chronic hypoxia, like heart failure or hypertrophy, prolongs the action potential due to downregulation of the transient outward K^+^ channels, predisposing the myocardium to delayed repolarization, calcium overload, and arrhythmias [20].

Two primary mechanisms lead to sarcoplasmic reticulum (SR) Ca^2+^ overload in acute ischemia:

1. Intracellular Na^+^ rise, due to reduced Na^+^/K^+^ ATPase activity and NHE stimulation by acidosis, inhibits Ca^2+^ extrusion via the NCX.
2. Sympathetic hyperactivation, common in ischemia, enhances β-adrenergic signaling and cAMP-mediated L-type Ca^2+^ currents (ICa), further loading the SR [21].

An overloaded SR is prone to spontaneous diastolic Ca^2+^ release via ryanodine receptor 2 (RyR2), triggering delayed afterdepolarizations (DADs) through the electrogenic NCX and potentially initiating arrhythmias. Early afterdepolarizations (EADs), occurring during repolarization, are another arrhythmogenic mechanism in repolarization-prolonging conditions [22]. Pharmacological agents, such as β-blockers (e.g., bisoprolol and metoprolol), mitigate these effects by attenuating ICa and the sympathetic drive, reducing post-infarction arrhythmic risk [23]. Intracellular acidosis, another hallmark of ischemia, impairs contractility by competing with Ca^2+^ at the troponin C binding site. Although transporters like NHE and the Na^+^/HCO_3_^−^ symporter buffer pH, their capacity is overwhelmed during ischemia, contributing to mechanical failure [24,25]. If ischemia persists, cells inevitably undergo death via multiple mechanisms, with necrosis and regulated necrosis (such as necroptosis) being predominant [26,27].

### 2.2. The Paradox of Reperfusion Injury

**Figure 2:**
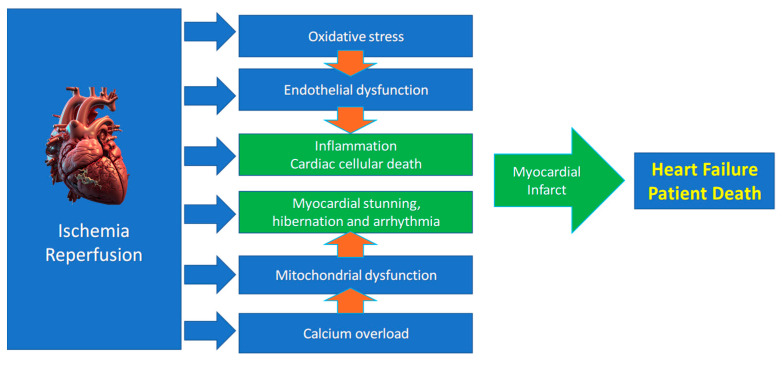
Main mechanisms of ischemia/reperfusion injury leading to heart failure.

While restoring oxygen is essential, reperfusion paradoxically amplifies injury via several mechanisms (Figure 2), including two major mechanisms:

(a) Rapid correction of acidosis via NHE leads to a sudden intracellular Na^+^ rise. If Ca^2+^ overload persists, this restoration of contractility can precipitate hypercontracture, damaging the cytoskeleton and membranes and causing necrosis and other forms of cell death [24,25,26,27,28]. Inhibiting NHE with agents like amiloride delays the normalization of pH and contractility, affording time for Ca^2+^ homeostasis restoration and reducing reperfusion injury in preclinical models [29]. Also, cardiac postconditioning (PostC) delays pH normalization [24,30], and acidic infusion in early reperfusion recapitulates many aspects of PostC [24,31,32].
(b) Oxidative stress, driven by a burst of reactive oxygen species (ROS) and reactive nitrogen species (RNS) at reperfusion, damages membranes and ion channels. Free radicals such as superoxide (O_2_^−^) and peroxynitrite (ONOO^−^) contribute to arrhythmogenesis and cell death [33]. However, ROS/RNS are essential to induce pre- and postconditioning cardioprotection [32,34,35,36,37].

Our understanding of the mechanisms driving reperfusion injury has advanced considerably thanks to research on conditioning mechanisms. We learned that reperfusion injury pathogenesis arises from the convergence of several pathways, including ion channel dysregulation, oxidative stress, mitochondrial dysfunction, and inflammation, leading to myocardial and endothelial dysfunction [6,38]. As mentioned, cells can die by multiple mechanisms in IRI. Cardiac cells that do not die can be stunned (so-called myocardial stunning) [39].

### 2.3. Myocardial Stunning

Following ischemia, even after blood flow is restored, contractile dysfunction can persist, a condition known as myocardial stunning. Despite adequate Ca^2+^ transients and preserved energy stores, the heart’s ability to contract remains temporarily impaired. This reversible dysfunction is not caused by permanent cellular damage but by a transient disruption in the responsiveness of the contractile machinery, particularly the myofilaments [39,40]. A central mechanism involves post-translational modifications, especially the degradation of troponin I (TnI), a key regulator of calcium sensitivity in contractile proteins, which ultimately reduces myofilament responsiveness to Ca^2+^. Another mechanism includes the dephosphorylation of phospholamban [41]. Nevertheless, the interplay between ischemic conditioning and myocardial stunning has remained a subject of debate [42,43].

### 2.4. Mitochondria and Mitochondrial Permeability Transition Pore: Gatekeepers of Cell Fate

Mitochondria play a dual role in myocardial IRI, both as targets and amplifiers of injury and as major players in cardioprotection. During ischemia, ATP depletion and metabolite accumulation sensitize mitochondria to reperfusion-induced ROS/RNS bursts. Complexes I and III of the electron transport chain are key ROS sources. Reperfusion triggers opening of the mitochondrial permeability transition pore (mPTP), leading to mitochondrial depolarization, ATP depletion, swelling, and eventual cell death [12]. Distinct modulation of F0F1 ATP synthase activity by IPC has implications for mPTP regulation [44,45]. Our group has demonstrated that targeting mitochondrial dysfunction offers therapeutic potential. For instance, nitroxyl (HNO) donors, such as IPA/NO and Angeli’s salt, limit infarct size and preserve mitochondrial integrity via a PKCε-dependent, KATP-independent mechanism [46]. Indeed, this cardioprotective pathway has been described by many other authors. In these studies, PKCε has emerged as one of the main mediators of mitochondrial protection during ischemic stress. Once activated, this isoform can translocate to mitochondria and modulate the opening of mPTP, thereby reducing cellular damage [47,48,49]. The PKCε mitochondrial import appears to be HSP90-dependent [50], and once inside the mitochondrial matrix, PKCε can phosphorylate ALDH2, contributing to the activation of mitochondrial KATP channels [51,52,53] and the modulation of oxidative stress [54,55]. PKCε has also been implicated in the regulation of apoptotic pathways, for instance, through phosphorylation of the BAD protein, which prevents outer mitochondrial membrane permeabilization [56], although this effect has not been definitively confirmed in the heart. In addition to being recognized as one of the key effectors of IPC-induced cardioprotection [48,57], selective activation of PKCε has proven effective in reducing infarct size and the incidence of ventricular fibrillation during reperfusion in animal models [58].

Of note, activation of PKCε has been shown to increase the expression of Kir6.2, the pore-forming subunit of KATP channels, thereby enhancing channel activity [59]. In the heart, the ATP-binding cassette protein SUR2A serves as a key regulatory subunit of KATP channels, forming functional pores through its association with Kir6.2 [60]. Overexpression of SUR2A confers robust protection against IRI and hypoxia/reoxygenation injury, as demonstrated by Jovanović’s group in both in vitro and in vivo models, primarily via an increase in KATP channel density at the sarcolemma [61]. One of the principal upstream regulators of SUR2A expression appears to be AMP-activated protein kinase (AMPK), whose activation by either hypoxia in mice or the direct AMPK activator 5-aminoimidazole-4-carboxamide riboside (AICAR) in H9c2 cells leads to upregulation of SUR2A levels [62]. Intriguingly, AMPK is a pivotal regulator of cardioprotective mechanisms [63,64]. Furthermore, physical exercise has been shown to increase SUR2A expression, contributing to improved cardiac Ca^2+^ homeostasis and enhanced myocardial resistance to stress [65]. In contrast, aging is associated with a marked decline in SUR2A expression [66], which may partly explain the reduced effectiveness of cardioprotective strategies in the elderly population [67]. Collectively, these findings underscore the central role of SUR2A in endogenous cardioprotection and support its potential as a therapeutic target in cardiovascular disease [60].

### 2.5. Redox Imbalance and Intracellular Signaling

Ischemia/reperfusion causes profound redox perturbations, influencing multiple signaling cascades. Our investigations into dysoxia reveal that fluctuating oxygen levels, more than sustained hypoxia, disrupt redox homeostasis and exacerbate injury [68]. Beyond direct ROS damage, redox-sensitive pathways modulate kinase/phosphatase activity, transcription, and translation. For instance, we show that oxidative stress impairs neuregulin/ErbB3 signaling, whereas PostC preserves ErbB3 protein synthesis and mitigates mitochondrial dysfunction [69].

Notably, ROS/RNS are not solely detrimental. In our seminal work [34], we established that redox signaling is essential for the cardioprotection elicited by ischemic PostC. This was highlighted in a commentary by Downey and Cohen, aptly titled “A really radical observation” [70]. Subsequent studies confirmed that acidosis, redox modulation, and delayed mPTP opening during early reperfusion are critical to successful PostC [32,71,72]. These findings underscore the delicate balance between harmful and protective roles of ROS/RNS, with therapeutic implications for modulating reperfusion injury and improving clinical outcomes after myocardial infarction. Nevertheless, ROS production increases significantly minutes after the beginning of the reperfusion phase, leading to oxidative stress and cellular damage. Multiple sources of ROS during reperfusion after myocardial ischemia include, but are not limited to, mitochondria, xanthine oxidoreductase, uncoupled nitric oxide synthase, and nicotinamide adenine dinucleotide phosphate (NADPH) oxidase [73].

### 2.6. Apoptosis and Prosurvival Signaling

Apoptosis is a prominent mode of cell death in IRI. Our data from several models indicate that infarct-sparing interventions are often associated with increased expression of anti-apoptotic proteins (e.g., Bcl-2, Pim-1), decreased cytochrome c release, and enhanced phosphorylation of survival kinases, such as Akt and extracellular signal-regulated kinase 1/2 (ERK1/2) [74,75]. Intriguingly, apelin, a peptide hormone, and its receptor APJ play a crucial role in several conditions, including protecting cells against IRI [76]. Indeed, apelin and its APJ receptor were shown to activate the PI3K–Akt–nitric oxide (NO) synthase (NOS), endothelial (eNOS) pathway via epidermal growth factor receptor (EGFR) and Src transactivation, providing protection only when administered during reperfusion as a form of pharmacological PostC [77,78]. This time-dependent efficacy reflects the dynamic expression of APJ receptor post-ischemia and underscores the importance of temporal precision in therapeutic interventions.

### 2.7. Connexin-43 and Electrical-Metabolic Coupling

Connexin-43 (Cx43), a key component of cardiac gap junctions, is the predominant connexin isoform expressed in ventricular cardiomyocytes, where it plays a crucial role in maintaining electrical and metabolic coupling through the formation of gap junctions. These intercellular channels are assembled from hemichannels—each composed of six Cx43 subunits—on adjacent cells, allowing the rapid exchange of ions and small signaling molecules necessary for synchronized cardiac contraction. Beyond its well-established role at the plasma membrane, Cx43 also localizes to mitochondria, where it contributes to key aspects of mitochondrial function, including oxygen consumption and potassium homeostasis [79]. Notably, the expression and distribution of both sarcolemmal and mitochondrial Cx43 are profoundly affected in various cardiac pathologies, such as hypertension, left ventricular hypertrophy, hypercholesterolemia, IRI, post-infarction remodeling, and heart failure [80]. Accumulating evidence, emerging from many laboratories, points to mitochondrial Cx43 as a critical mediator of cardioprotective signaling, particularly in the context of ischemic conditioning. We explored the role of Cx43 in IPC and PostC protocols. Cx43 phosphorylation was found to correlate with improved cardiac recovery and infarct limitation, possibly by maintaining mitochondrial function and reducing electrical uncoupling during reperfusion injury [75,78].

Understanding the multifaceted roles of Cx43, and especially its mitochondrial functions, is essential for developing novel therapeutic strategies aimed at preserving cardiac function under stress conditions.

## 3. Endogenous and Pharmacological Cardioprotection

**Figure 3:**
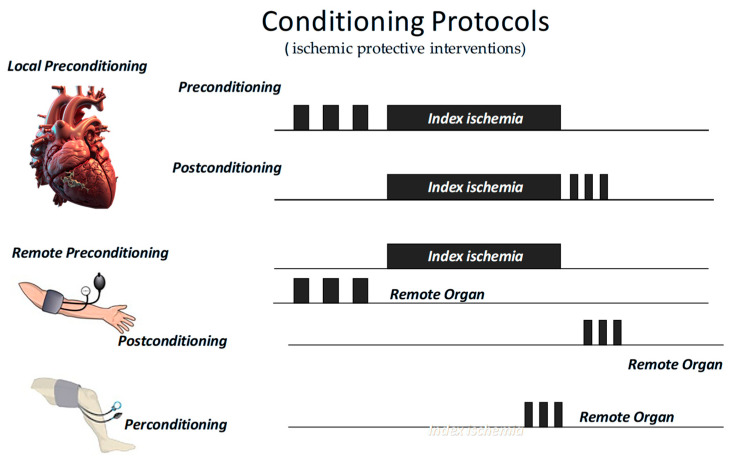
Short periods of ischemic protective interventions may be applied before (preconditioning) or after (postconditioning) an index ischemia, either locally or remotely. In the latter case, they may also be applied during the index ischemia (perconditioning).

As we said, most of our knowledge in this field started with the discovery of IPC, defined as brief episodes of sublethal ischemia followed by reperfusion, which can render the myocardium more resistant to subsequent prolonged ischemic insults. This protection involves both mitochondrial and redox-dependent signaling [81]. Now, we know that cardioprotection can be achieved through both ischemic conditioning protocols and pharmacological activation of endogenous prosurvival pathways. These two approaches share molecular targets, including the inhibition of apoptotic signaling, preservation of mitochondrial integrity, and maintenance of endothelial function. Among conditioning strategies, IPC has been shown to delay infarct progression, primarily by protecting against reperfusion injury [82]. Ischemic PostC, administered at the onset of reperfusion through short cycles of re-occlusion, similarly limits reperfusion-induced myocardial damage. Remote ischemic preconditioning, achieved by inducing transient ischemia in a distant organ such as a limb, can also confer systemic cardioprotective effects (Figure 3) [83,84].

All these “ischemic protective interventions” activate similar, though not identical, coordinated cascades of intracellular signals, beginning at the sarcolemma and progressing through cytosolic kinase pathways to ultimately stabilize mitochondria and inhibit cell death (Figure 1). Therefore, studies conducted over recent years suggest that ischemic conditioning is not merely an experimental procedure but rather a systemic biological defense mechanism against irreversible tissue damage. Naturally, any short protective ischemia can act both locally and remotely in early and delayed phases, involving rapid molecular signals and transcriptional modifications, as well as a complex interplay between the autonomic nervous system, the immune system, and the circulation [14,85,86,87]. Therefore, we can infer that the traditional distinction between the types and timing of conditioning is mainly artificial, as all forms activate similar and integrated protective programs. A broader understanding of this phenomenon could enhance its clinical application.

Although various pharmacological mimetics targeting cardioprotective signaling pathways have been developed and tested in preclinical models, their translation into consistent clinical benefit has proven challenging [88,89,90,91,92,93,94]. These mimetics comprise a wide range of endogenous and pharmacological agents, which we cannot examine in detail here. They can be broadly classified into three categories:

(a) Agents that activate cardiomyocyte surface receptors, such as adenosine, bradykinin, and glucagon-like peptides 1 and 2 (GLP-1, GLP-2) [95,96,97];
(b) Compounds acting on intracellular signal transduction pathways, such as STAT3 or PKC activators [58,98,99];
(c) Agents targeting mitochondrial mechanisms, such as mitochondrial KATP channel openers and mPTP inhibitors [100,101,102].

While some compounds (e.g., cyclosporine A, aimed at inhibiting mPTP opening) initially showed promise in early-phase clinical trials [102], their efficacy could not be confirmed in larger studies [103]. Conversely, drugs such as ticagrelor, although not originally developed as cardioprotective mimetics, have shown potential cardioprotective effects by modulating similar signaling pathways [104,105] (also see below). This overlap may partly explain the observed inconsistencies: the activation of shared protective pathways by standard-of-care medications can mimic conditioning and can mask or limit the additional benefit of other specific mimetics. Moreover, clinical variability in timing of administration, dosing, patient comorbidities, and interactions with concomitant therapies further complicate the reproducibility of cardioprotective effects observed in preclinical settings. Notably, biological sex is increasingly recognized as a critical determinant of cardioprotective responses, underscoring the need for personalized approaches in cardiovascular therapy. Nevertheless, the role of sex as a biological variable in the conditioning context remains controversial [106] (also see below).

### 3.1. Preconditioning, Postconditioning, and Kinase Signaling

As we said, IPC and PostC trigger cardioprotection via the activation of similar pathways, and these have been named NO/protein kinase G (PKG) signaling, reperfusion injury salvage kinase (RISK), and survivor activating factor enhancement (SAFE) pathways. These involve PI3K/Akt, ERK1/2, signal transducer and activator of transcription 3 (STAT3), PKC, and glycogen synthase kinase-3 beta (GSK-3 beta), which converge on mitochondrial targets, such as mPTP, and pro-apoptotic proteins, like Bax, with differences among species [6,98].

Several additional pathways and mechanisms have been implicated in the heart’s response to ischemia/reperfusion. Challenging and conditioning strategies, especially those involving mitochondrial integrity, metabolism, and inflammation, contribute substantially to cardioprotection and may become targets for novel therapies [107,108,109,110,111]. The emerging evidence highlights the Wnt signaling pathway as a critical, yet underexplored, regulator of IRI. Acting through complex interactions, Wnt signaling interfaces with PI3K/Akt and other multiple pathways, including Notch, transforming growth factor beta (TGF-β), nuclear factor kappa-light-chain-enhancer of activated B cells (NF-κB), bone morphogenetic proteins (BMPs), N-methyl-D-aspartate receptor (NMDAR)/Ca^2+^/Activin A, Hippo/yes-associated protein (YAP), toll-like receptors 4 (TLR4)/TRIF, and hepatocyte growth factor (HGF)/c-Met. These interactions orchestrate a variety of pathological and reparative processes, such as apoptosis, inflammation, oxidative stress, extracellular matrix remodeling, angiogenesis, hypertrophy, fibrosis, and ferroptosis. Wnt signaling represents an integrative network capable of modulating both injury and repair in IRI settings. A dualistic role of canonical versus non-canonical Wnt activation has been reported and proposed as cardioprotective paradigms [112], where canonical Wnt tends to promote tissue recovery, while non-canonical activation is often deleterious.

As mentioned earlier, pharmacological approaches that mimic these protective signaling cascades have also been explored, although clinical trials have yet to demonstrate consistent prognostic benefit (see below). Pharmacological and endogenous agents such as platelet-activating factor (PAF) and apelin-13 have been shown to modulate the response to IRI. PAF, a phospholipid mediator released by an ischemic–reperfused heart, contributes to myocardial IRI development [113] but also plays a role in IPC cardioprotection via activation of the PKC/Akt/NOS pathway [114,115]. PAF pretreatment promotes cardioprotection by inactivating GSK-3β and, thus, promoting mPTP closure during reperfusion [116,117]. Like other mediators, including ROS, PAF exerts dual effects, which can be either deleterious or protective depending on concentration and timing of release. The importance of timing is also evident for apelin-13, which does not mimic preconditioning but reproduces postconditioning effects when administered during early reperfusion. As above mentioned, this effect is mediated by the activation of PI3K-Akt-NO signaling and inhibition of phosphatase and the tensin homolog (PTEN) via Src-mediated phosphorylation [77,78]. EGFR transactivation triggered by apelin underscores the complexity of G-protein coupled receptor (GPCR) crosstalk in cardioprotection. In particular, the selective cardioprotective effect of apelin during the reperfusion phase highlights some mechanistic differences between preconditioning and postconditioning [118,119,120]. Moreover, pharmacological cardioprotection with drugs like ticagrelor or NOD-like receptor family pyrin domain-containing protein 3 (NLRP3) inhibitors has also been shown to activate the RISK cascade and modulate redox balance, although our results indicate that these effects are not additive, highlighting potential pathway saturation or competition among different pathways [121].

Although platelets are central mediators of acute coronary syndromes, where their activation and aggregation drive the onset and progression of IRI, the emerging evidence indicates that they can also contribute to cardioprotection [122,123]. Indeed, platelets from healthy individuals are capable of triggering prosurvival signaling cascades, including the RISK pathway. However, studies investigating the effects of aspirin or ticagrelor, both in local and remote ischemic conditioning, have introduced further complexity, casting uncertainty on the precise role of platelets in cardioprotective mechanisms [124,125]. Further studies are necessary to ascertain the precise role of platelets.

Again, it is also important to recognize that the effectiveness of cardioprotective interventions and drugs is modulated by multiple factors, including patient age, sex, comorbidities, concomitant medications, and procedural aspects, such as the type of revascularization, the method used to assess cardiac injury, and adherence to therapies. Understanding and overcoming these confounders remains a critical step in translating cardioprotection into routine clinical practice.

### 3.2. The Role of Gasotransmitters

Nitric oxide, carbon monoxide (CO), and hydrogen sulfide (H_2_S) act as powerful endogenous mediators of cardioprotection. NO promotes vasodilation, inhibits platelet aggregation, and regulates mitochondrial respiration. CO exerts similar effects, while H_2_S primarily improves mitochondrial bioenergetics and reduces oxidative stress [91,92,125].

Our review on NO and CO interactions with platelets in cardioprotection [126] emphasized their dual effects on both vascular tone and platelet activity, suggesting a concerted role in reducing IRI. Interestingly, the modulation of platelet-derived extracellular vesicles (EVs) by these gasotransmitters may represent a novel mechanism of remote signaling in cardioprotection, which requires further investigation. We briefly mention the role of gaseous agents in cardioprotection, given their relevance, and refer the interested reader to recent comprehensive reviews on the topic, e.g., [90,92,127].

### 3.3. Sex Differences in Cardioprotection: A Neglected Variable in the Presence of Comorbidities

Among the various factors influencing cardioprotection, the evidence suggests that sex hormones and sex-specific signaling pathways play a significant role in modulating its efficacy in the presence of comorbidities. Using female spontaneously hypertensive rats, our group showed that activation of the G protein-coupled estrogen receptor (GPER) by G1 elicits infarct size reduction and post-ischemic functional recovery through Notch1 and PI3K-Akt signaling—effects abolished by GPER inhibition [128]. Moreover, these effects persisted despite hypertension, a comorbidity known to blunt ischemic conditioning, suggesting that female-specific signaling confers unique cardioprotective advantages. Also, PostC cardioprotection is influenced by sex [129]. The cardiomyocyte-specific knockout of monoamine oxidase B (MAO-B) reduced myocardial infarct size and mortality after the ischemia/reperfusion protocol in male mice but had no protective effect in females, which exhibited lower infarct sizes in wild-type controls [130]. These findings are particularly relevant considering the underrepresentation of female models and patients in cardioprotection research. It reinforces the need for sex-stratified preclinical and clinical studies to fully harness cardioprotective therapies. Findings provide evidence that the female myocardium may exhibit greater plasticity in engaging prosurvival signaling pathways in response to both ischemic and pharmacological stimuli, especially in the presence of comorbidities. For instance, a study in diabetic mice subjected to transient middle cerebral artery occlusion showed that male diabetic mice exhibited reduced capillary reperfusion and larger cerebral infarcts compared to controls, whereas diabetic females did not differ significantly from their controls. Premenopausal females appear to be protected against diabetes-related capillary dysfunction and brain injury [131]. This observation supports the development of sex-specific cardioprotective strategies that consider biological sex as a critical determinant of therapeutic efficacy [132,133]. However, it is important to acknowledge that studies employing models more closely resembling human physiology (pigs) have not consistently confirmed a significant role for sex per se, attributing observed differences to variations in myocardial mass [106]. This aligns with our previous work demonstrating that pharmacologically induced cardiac hypertrophy leads to a transition from a cardioprotected state to a maladaptive and ischemia-susceptible phenotype, highlighting the influence of structural features on cardioprotective capacity [134,135]. Although some findings indicate that comorbidities can differentially affect IRI between sexes, this field remains underexplored. Most preclinical studies focus on healthy animals, often neglecting sex and comorbidity influences and very often neglecting the combination of sex and comorbidities. Incorporating both sexes and relevant comorbidities in experimental models is essential for a comprehensive understanding of biological responses to ischemia/reperfusion.

## 4. Inflammation and the NLRP3 Inflammasome

While IRI has traditionally been interpreted through the lens of oxidative stress, calcium overload, and metabolic dysfunction, it is now well established that sterile inflammation, particularly via innate immune activation, plays a central pathogenic role. Among the mediators of this inflammatory cascade, the NLRP3 inflammasome has emerged as a key integrator of mitochondrial stress, redox imbalance, immune signaling, and metaflammation in IRI and metabolic syndrome [99,136]. Several recent findings on NLRP3 activation in metabolic syndrome provide evidence of the inflammasome’s central role in disease progression and organ dysfunction and the target tissues of metaflammation, in particular, in cardiovascular, hepatic, and renal complications, with a focus on oxidative stress, advanced glycation end-products (AGEs), and inflammation in the field of myocardial IRI [132].

### 4.1. NLRP3 Inflammasome Activation in Cardiomyocytes and Macrophages

**Figure 4:**
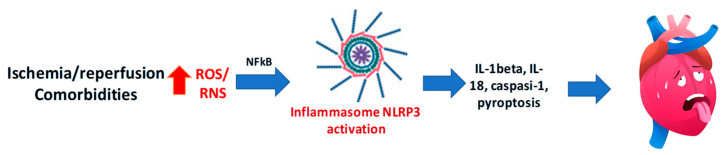
Excessive reactive oxygen species (ROS) and reactive nitrogen species (RNS) are key triggers of NLRP3 inflammasome activation, leading to caspase-1 activation, IL-1β and IL-18 release, and pyroptosis, thus contributing to myocardial ischemia/reperfusion injury.

The NLRP3 inflammasome is a multiprotein complex that, upon activation by multiple mechanisms, including mitochondrial-derived ROS and RNS, ion fluxes, or the so-called damage-associated molecular patterns (DAMPs), promotes the cleavage of pro-caspase-1 into its active form. This process leads to the maturation and release of interleukin (IL)-1β (IL-1β) and IL-18 and the induction of pyroptosis, a proinflammatory form of programmed cell death (Figure 4). Foundational studies by Abbate and Toldo first established the centrality of NLRP3 in myocardial injury [137].

Our group has further delineated the cell-specific roles of NLRP3 in the heart, showing that both cardiomyocytes and cardiac macrophages contribute to inflammasome-driven damage. We demonstrated that the selective NLRP3 inhibitor INF150 confers protection in macrophages but fails to reduce infarct size in the whole heart due to poor cardiomyocyte uptake [75]. Interestingly, its precursor, INF195, is cardioprotective at low doses, as it is capable of penetrating cardiomyocytes where it is metabolically converted to INF150 [138]. These findings underscore a major translational challenge: the importance of pharmacokinetics and cell-specific delivery in the development of inflammasome-targeted therapies [136,139].

### 4.2. Redox Crosstalk and the Inflammatory Loop

The relationship between oxidative stress and NLRP3 activation is bidirectional. Mitochondrial ROS serve as upstream activators of the inflammasome, while NLRP3 signaling, in turn, promotes further ROS generation, thus sustaining a vicious cycle of redox stress and inflammation, leading to cell death. Our studies have shown that inhibition of either ROS production or NLRP3 activity leads to mutual downregulation, providing a strong rationale for dual-target pharmacological strategies in myocardial IRI [139].

#### 4.2.1. Metabolic Comorbidities and NLRP3 Exacerbation

Diet-induced models of metabolic stress offer further insight into the interaction between inflammasome signaling and comorbid conditions. In mice subjected to a high-fat, high-fructose (HFHF) diet, we observed increased infarct size, elevated NLRP3 expression, enhanced caspase-1 activation, and suppression of protective RISK and HIF-2α pathways [140]. These findings were corroborated in a rat model of high-fat diet-induced metaflammation [141]. These results align with the concept of “hidden comorbidities” as covert metabolic alterations that blunt the heart’s ability to engage endogenous protective mechanisms [15,142,143].

#### 4.2.2. Translational Challenges and Perspectives for NLRP3 Targeting

Despite strong preclinical evidence, the clinical translation of NLRP3-targeted therapies has been limited. Reviews by our group and others on this topic have identified critical barriers: animal models often lack advanced age, sex diversity, comorbidities, and multidrug therapy typical of patients with acute coronary syndromes. Moreover, endpoints such as infarct size are emphasized over clinically meaningful outcomes like mortality, remodeling, or arrhythmias. Yet, drug distribution at the tissue and cellular level is rarely considered [144,145,146]. Cross-talk between NLRP3 and other pathways and factors, such as chromogranin A and its derivatives, may modulate inflammation and cell death mechanisms relevant to cardioprotection and deserves further consideration [147,148,149].

The contrast in activity between INF150 and INF195 reinforces the need for strategies that ensure effective myocardial delivery. Future cardioprotective interventions will require multi-target compounds or combinatorial regimens that address both inflammation and redox imbalance, with specific attention to cellular compartmentalization and disease context. Studies on INF150 and INF195 have highlighted the crucial role of different cell types within a given organ. Ongoing experiments from our group (unpublished observations) suggest that INF195 shows greater efficacy in protecting endothelial cells exposed to various stressors. This finding is particularly relevant considering the central role of vascular protection in preserving organ function in the context of IRI [150].

## 5. Comorbidities in Cardioprotection and the Impact of Metabolic Syndrome: Diabetes, Obesity, and Metainflammation

**Figure 5:**
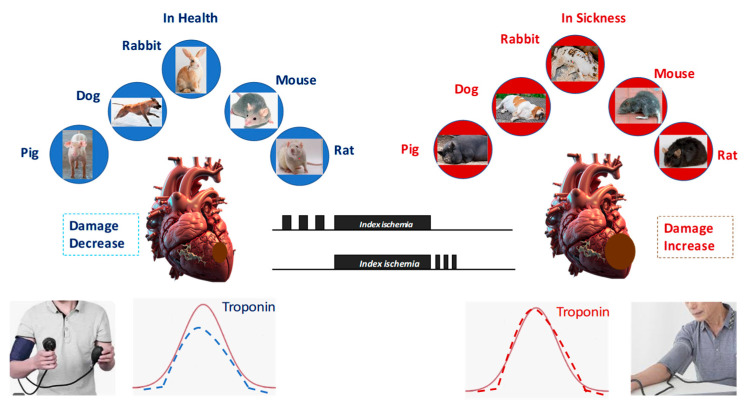
All forms of conditioning protect against ischemia/reperfusion injury in all species studied. However, in the presence of comorbidities and comedications, conditioning protocols lose their protective effect.

Metabolic syndrome (MetS), a cluster of conditions including abdominal obesity, dyslipidemia, hypertension, metainflammation, and glucose intolerance, represents a multi-pronged assault on cardioprotective mechanisms. The promise of cardioprotective strategies observed in preclinical models has too often failed to translate into meaningful clinical benefits. A major factor limiting success is the presence of comorbidities, such as diabetes mellitus, obesity, metainflammation, and metabolic syndrome, which profoundly alter myocardial signaling and cellular responses to stress and protection. These conditions not only exacerbate IRI but also blunt the efficacy of cardioprotective interventions, including ischemic and pharmacological conditioning (Figure 5).

### 5.1. Diabetes and the Resistant Myocardium

Diabetes mellitus alters cardiac metabolism, increases oxidative stress, and disrupts endothelial and platelet function, creating a hostile environment for protective signaling [121,151]. Peter Ferdinandy and colleagues described the “diabetic heart” as intrinsically less responsive to IPC and pharmacological cardioprotective stimuli due to alterations in key kinases, mitochondrial function, and microvascular integrity [152]. This concept was developed in a couple of reviews in collaboration with the authors of *Cost Action in Cardioprotection* [142,153].

In models of insulin resistance and type 2 diabetes, the protective effects of conditioning were largely abolished, with NLRP3 inflammasome overexpression, increased caspase-1 activation, and impaired PI3K-Akt signaling [136,140]. These maladaptations led to larger infarcts and greater cell death. Moreover, our work showed that ErbB3 upregulation, a stress response beneficial in healthy myocardium, fails to preserve its protein expression in diabetic ischemia/reperfusion hearts unless rescued by PostC strategies [69].

### 5.2. Obesity and the “Obesity Paradox”

Obesity is associated with low-grade chronic inflammation (“metaflammation”), increased ROS, endothelial dysfunction, and altered cardiometabolic responses. Intriguingly, clinical observations sometimes suggest better outcomes in obese heart failure patients, a phenomenon dubbed the “obesity paradox”. However, our group demonstrated that in models of myocardial IRI, obesity correlates with reduced sensitivity to conditioning protocols and increased vulnerability to NLRP3-mediated damage [154]. This supports the notion that while obesity may sometimes mask the clinical severity of disease, it undermines cardioprotective signaling. Notably, we found that obese animals had altered mitochondrial substrate utilization and blunted activation of RISK kinases, further impairing the recovery phase post-IRI.

### 5.3. Metainflammation and Multilevel Disruption

The concept of “metaflammation”—the metabolic priming of innate immunity—has gained traction, reinforcing the idea that cardioprotection in MetS must address not only cardiac cells but also systemic immune–metabolic interfaces. In our studies, metainflammation and induced upregulation of the NLRP3 inflammasome in multiple tissues—heart, liver, and kidney—are considered in the light of oxidative stress, AGEs, and endothelial dysfunction [136]. In our studies, we considered that systemic inflammatory state disrupts not only local myocardial defense but also conditioning signals mediated by extracellular vesicles and humoral factors [120,155,156].

### 5.4. Comorbidities and Cardioprotection

Importantly, our studies on female hypertensive models suggest that sex may modulate the impact of comorbidities. While hypertension typically abrogates ischemic conditioning responses, female rats treated with GPER agonists retained Notch1- and PI3K-mediated cardioprotection, despite elevated blood pressure [128]. This implies that sex-specific hormonal and genetic factors may offer residual protective capacity, even in high-risk settings. Rainer Schulz and Peter Ferdinandy have strongly advocated for a shift toward comorbidity-inclusive models in cardioprotection research [15]. In our collaborative works [142,144], we emphasize that most translational failures stem from neglecting the altered molecular landscape of diseased hearts. We urge researchers to adopt models incorporating diabetes, aging, and obesity as the new gold standard rather than the exception when studying cardioprotection in both sexes.

## 6. Extracellular Vesicles and Conditioning: Systemic Mediators of Cardioprotection

Remote ischemic conditioning (RIC), wherein brief ischemic episodes in distant tissues protect the heart from subsequent IRI, has revealed the crucial role of blood-borne mediators in cardioprotection. Among these, extracellular vesicles (EVs)—nano- to micro-sized lipid bilayered particles released from various cell types—have emerged as key vectors of intercellular communication. They transport proteins, lipids, mRNAs, and non-coding RNAs capable of reprogramming recipient cells under stress [157]. Our group has demonstrated that a proinflammatory environment can modify the cardioprotective activity of EVs, as demonstrated in endothelial-derived EVs released in response to the proinflammatory cytokine interleukin 3 (IL3 [158]).

### 6.1. EVs as Vectors of Cardioprotective Signals

In a collaborative study, it has been shown that blood-derived EVs, isolated from patients with acute coronary syndrome (ACS) undergoing percutaneous coronary intervention (PCI), carry functional signals capable of modulating ischemia/reperfusion responses in vitro and ex vivo [159]. Notably, these EVs exhibit cardioprotective activity that correlates with the clinical outcome and timing of PCI, suggesting their role as both biomarkers and effectors of myocardial protection.

We also showed that the cardioprotective potential of circulating EVs is modulated by disease state. In patients with ST-elevation myocardial infarction (STEMI), the cargo of plasma-derived EVs differs significantly compared to healthy controls, particularly regarding microRNAs and stress-related proteins. These EVs affected infarct size and cardiomyocyte viability in murine Langendorff models, establishing a functional readout of their protective efficacy [160].

### 6.2. Endothelial-Derived EVs: Angiogenic and Anti-Inflammatory Signals

The endothelium is a central player in RIC and EV-mediated cardioprotection. Our studies showed that endothelial cells subjected to simulated ischemic PostC released EVs enriched in angiogenic factors and microRNAs, such as miR-21 and miR-126, which enhanced capillary formation and reduced apoptosis in recipient cardiac cells [74]. These EVs also carried modulators of the PI3K-Akt and eNOS pathways, mimicking the effects of pharmacological PostC.

Such findings are consistent with the broader literature, including work by Ferdinandy and colleagues, who propose that EV cargo content is an “integrated readout” of the conditioning phenotype and may represent an ideal tool for therapy or patient stratification [161,162].

### 6.3. Influence of Comorbidities on EV Function

One of our most insightful observations is that EV composition and function are profoundly affected by comorbidities. In models of diabetes and obesity, both the release and cardioprotective efficacy of EVs are impaired. This includes reductions in anti-apoptotic miRNAs, altered surface marker expression, and blunted activation of survival pathways in recipient cells. These findings are particularly relevant given that most patients eligible for RIC or PCI have metabolic syndrome or insulin resistance [155,156]. Interestingly, EVs from obese and diabetic individuals or carrying hypercholesterolemia may even carry proinflammatory cargo, promoting maladaptive remodeling pre- and post-IRI. This suggests that in certain contexts, EVs can lose their protective function and become vectors of damage—a concept now gaining recognition in the field [155,156,157,162].

## 7. Challenges and Future Perspectives

The IMPACT studies—IMproving Preclinical Assessment of Cardioprotective Therapies—represent a significant challenge and a step forward in enhancing the rigor, reproducibility, and translational potential of cardioprotection preclinical research.

The IMPACT Pig Study [163] is a large, multicenter, randomized study that investigated the effect of IPC in a clinically relevant porcine model of acute myocardial infarction. Despite IPC’s robust efficacy in single-center settings, this study revealed substantial variability across centers. The findings underscore the importance of multicenter validation and highlight potential challenges in translating IPC into clinical practice. The IMPACT Rodent Study [81] is a study conducted in parallel using a small animal model (rats and mice), and this multicenter randomized, controlled trial also assessed IPC’s protective effects. The majority of sites demonstrated significant cardioprotection with IPC, underscoring the importance of tightly controlled experimental conditions to achieve consistent results.

Overall, the IMPACT initiative has redefined standards for preclinical cardioprotection studies, demonstrating that interventions like IPC may be effective when evaluated with multicenter, clinically reflective rigor. These results call for more robust and standardized protocols and suggest that reproducibility in multicenter settings should be a prerequisite before translating cardioprotective strategies into clinical trials.

Also, EVs represent a challenge that may require an approach similar to the IMPACT studies. EV-based therapies hold promise for delivering conditioning-like signals in high-risk patients who may not respond to conventional stimuli [134]. However, challenges remain: standardization of EV isolation, dose control, cellular origin, and route of administration are all unresolved. Moreover, the systemic inflammatory environment of patients with comorbidities must be considered when evaluating the efficacy of EV-based interventions.

Beyond the IMPACT initiative and EV-based strategies, several critical challenges remain in the cardioprotection field, including the direct contribution of NLRP3 inflammasome to cardiac dysfunction in the context of IRI.

### 7.1. Poor Translatability of Promising Preclinical Interventions

A major challenge is the poor translatability of promising preclinical interventions into effective clinical therapies. As mentioned, this poor translatability is often due to differences in considered patients, lack of standardization, and insufficient consideration of comorbidities, such as aging, diabetes, or inflammation. Several clinical trials have been completed or are currently ongoing, yielding mixed results [164].

***Completed Studies:*** Among the completed studies showing neutral or limited clinical benefit are those evaluating the use of drugs and conditioning procedures in this setting. For instance, several clinical trials have investigated adjunctive strategies to reduce infarct size in STEMI patients undergoing primary percutaneous coronary intervention (PPCI). The EARLY-BAMI trial [165] tested early intravenous metoprolol prior to PPCI and found only modest benefits, highlighting the need for larger studies. The COMBAT-MI trial combined RIC with exenatide, aiming for synergistic cardioprotection, but neither treatment—alone or combined—reduced infarct size [166]. In the CIRCUS trial, cyclosporine added to PPCI failed to improve one-year outcomes in anterior STEMI patients [103]. Similarly, the PITRI study found that pre-reperfusion cangrelor did not reduce infarct size or microvascular obstruction in patients already treated with oral ticagrelor, despite lowering platelet reactivity [167].

#### A Renewed Effort in Translational Cardioprotection

Some trials reflect a renewed effort to translate promising cardioprotective mechanisms into practice. Among these, clinical trials are exploring their potential in more targeted patient groups. For example, RIP-HIGH [NCT04844931] is investigating RIC in high-risk STEMI patients to determine whether pre-reperfusion conditioning can reduce short-term mortality and prevent heart failure. Meanwhile, RIC-AFRICA [NCT04813159] is a sham-controlled, randomized trial enrolling STEMI patients in sub-Saharan Africa (treated primarily with thrombolysis) and testing whether RIC applied daily before and after reperfusion lowers 30-day all-cause death and new-onset heart failure. These trials aim to assess whether targeting higher-risk populations, unlike previous large RIC studies in lower-risk patients, might reveal clinically meaningful benefits from this simple, low-cost intervention. Worthy of mention is the COOPERATION trial, which is evaluating the cardioprotective effects of dexmedetomidine (an α2-adrenergic receptor agonist) administered during PPCI in STEMI patients, with infarct size assessed by cardiac MRI as the primary endpoint [NCT04912518]. Interestingly, a clinical trial is currently assessing supersaturated oxygen (SSO_2_) therapy to optimize myocardial salvage in acute settings [NCT05790876]. The rationale for this approach has also been discussed in a recent review [16].

***EV and Exosome Delivery:*** Several preclinical studies have shown the cardioprotective potential of EVs and exosomes in in vitro studies and animal models of myocardial infarction, e.g., [155,156,157,158,159,160,161,162,168]. While clinical evidence remains limited, early trials have confirmed the safety of EV- and exosome-based therapies in humans. To the best of our knowledge, [NCT05669144] is the only ongoing clinical trial investigating mesenchymal stem cell-derived exosomes for myocardial infarction, and it is currently in the recruitment phase (status: unknown as of July 2025). This study aims to assess the safety and functional impact of intracoronary/intramyocardial exosome delivery during coronary artery bypass grafting (CABG) in patients with severe systolic dysfunction. In randomized controlled trials, we showed that EVs from ACS patients collected before PCI (EV-naive) were cardio-protective, while those obtained after PCI or following RIC lost this protective effect both in vitro and ex vivo [155,156].

***NLRP3 Inflammasome Inhibition***: Although no clinical trials have yet directly assessed NLRP3 inflammasome inhibition for reducing myocardial infarction, several anti-inflammatory and anti-atherosclerosis strategies have been explored. For instance, colchicine, a microtubule polymerization inhibitor, indirectly affects NLRP3 activation and has shown promising results in reducing cardiovascular events in trials such as LoDoCo2 [169] and COLCOT [170]. However, the COVERT-MI trial failed to demonstrate a reduction in infarct size or improvement in cardiac function with colchicine in an acute MI setting [171]. Anakinra, an IL-1 receptor antagonist, reduced inflammatory markers and heart failure-related outcomes in small studies, though the results have been mixed regarding cardiovascular events [172,173]. The CANTOS trial with canakinumab, a selective IL-1β inhibitor, provided robust evidence for the inflammatory basis of coronary artery disease, showing reduced cardiovascular events without affecting lipid levels but raised concerns about cost and infection risk [174]. Several trials are ongoing to further evaluate NLRP3 inflammasome inhibitors [173], including COACS (Colchicine for Acute Coronary Syndromes; NCT01906749), CLEAR SYNERGY (Colchicine and Spironolactone in Patients with MI/SYNERGY Stent Registry; NCT03048825), CONVINCE (Colchicine for Prevention of Vascular Inflammation in Noncardioembolic Stroke; NCT02898610), and REDHART2 (NCT03797001), a randomized, double-blind, placebo-controlled trial evaluating the effects of anakinra on peak aerobic exercise capacity.

Collectively, these ongoing preclinical and clinical studies underscore the critical importance of standardized multicenter designs, early intervention timing, and comorbidity-aware protocols in improving translational success. Future cardioprotective strategies will likely require integrative, multimodal approaches, leveraging both pharmacologic and non-pharmacologic mechanisms in well-characterized patient cohorts. Additionally, the identification of robust, clinically relevant biomarkers of cardioprotection is still lacking in preclinical studies. Future studies should embrace integrative approaches, combining multi-omics technologies, advanced in vivo imaging, and systems biology to better understand the complexity of cardioprotective mechanisms. Importantly, collaboration across centers and disciplines should become the norm rather than the exception in order to overcome variability and improve reproducibility. These elements, together, represent the future directions and challenges the field must address to move toward effective and individualized cardioprotective therapies.

## 8. Conclusions

While significant advances have recently been made in understanding the cellular mechanisms underlying ischemia and cardioprotection in relation to inflammasome inhibition and cross-talk between macrophages and cardiomyocytes, the translation of these findings into improved patient outcomes remains limited. Bridging this gap requires not only validation of preclinical targets but also a shift toward clinical endpoints that reflect true patient benefit, such as survival and long-term cardiac function.

The inconsistent findings from recent multicenter preclinical and clinical trials underscore the need for larger, well-designed studies to reliably assess the effectiveness of strategies targeting myocardial IRI. These include integrative approaches that reinforce the rationale for EVs as both biomarkers and effectors of cardioprotection, as well as the potential use of inflammasome inhibitors in various clinical contexts. Coordinated efforts across institutions will be crucial to overcome variability in protocols, patient populations, and outcome measures, facilitating the transition from proof-of-concept to clinical application.

Given the multifactorial nature of IRI and heart failure progression, single-target approaches are unlikely to suffice. The development of integrative biomarkers—reflecting inflammation, metabolism, and cell death—will facilitate patient stratification and treatment monitoring. Concurrently, multi-target therapies that modulate immune, neural, and cardiomyocyte responses may offer synergistic benefits and greater clinical efficacy.

Since comorbidities and human pharmacological therapies greatly influence IRI, future cardioprotective strategies must be tailored to high-risk populations, such as patients from underdeveloped countries, older adults, diabetics, and females with comorbidities, where conventional interventions are either not available or often fail. Personalized approaches based on genetic, metabolic, and immune profiles could maximize therapeutic efficacy and minimize adverse effects. With this precision-oriented mindset, the translation from bench to bedside may finally achieve its goal: durable cardiac protection and improved survival for patients at risk.
